# Supplementary material for: Neuroprotection of Andrographolide against Neurotoxin MPP+-Induced Apoptosis in SH-SY5Y Cells via Activating Mitophagy, Autophagy, and Antioxidant Activities
Source: Int J Mol Sci. 2023 May 10;24(10):8528. doi: 10.3390/ijms24108528 (PMC10217882; doi:10.3390/ijms24108528)
Supplement: Supplementary file 1 [file ijms-24-08528-s001.zip › ijms-2369718-supplementary.pdf]

Supplementary figures

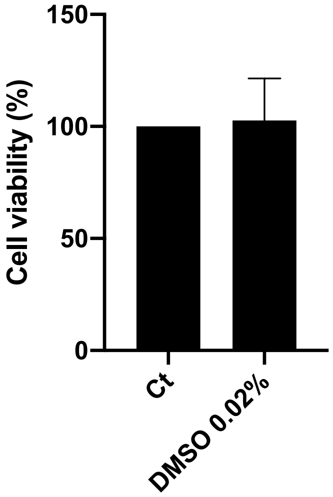

Supplementary figure S1. Cytotoxicity effect of DMSO on SH-SY5Y cells. Cell viabilities were evaluated by MTT assay. SH-SY5Y cells were treated with 0.02% of DMSO for 24 h.

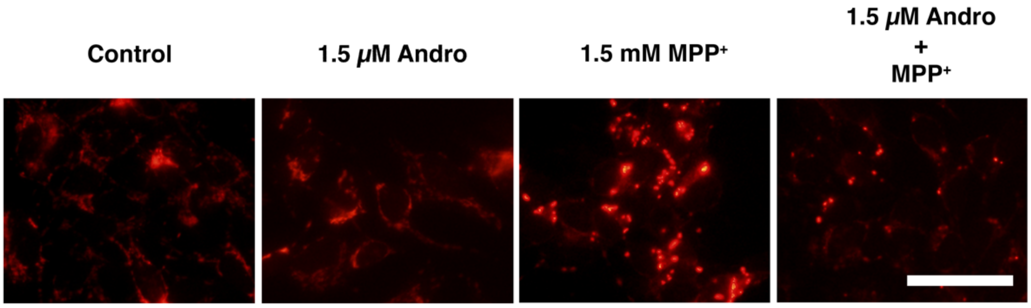

Supplementary figure S2. Protective effect of Andrographolide against MPP^+ induced neuronal death by enhancing autophagy and mitophagy induction. SH-SY5Y cells were incubated with or without 1.5 μM of Andro for 24 h followed by incubation of MPP^+ 1.5 mM for 16 h. Morphology of mitochondria stained with MitoTracker Red.
